# Gut microbial metabolism in aging: from metabolite production to organ-specific immunometabolic effects

**DOI:** 10.3389/fnut.2026.1984798

**Published:** 2026-10-15

**Authors:** Zhen Wang, Yang Liu

**Affiliations:** 1 Graduate School, Heilongjiang University of Chinese Medicine, Harbin, China; 2 Department of Critical Care Medicine, First Affiliated Hospital of Heilongjiang University of Chinese Medicine, Harbin, China

**Keywords:** aging, gut microbiota, gut-organ axis, immunometabolism, inflammaging, microbial metabolites, personalized nutrition

## Abstract

Age-related changes in gut microbiota metabolism are heterogeneous across populations and pathways, and their relationships with immunometabolism and organ phenotypes should be interpreted in the context of health status and disease. Microbial abundance or the concentration of a single metabolite does not, by itself, capture microbial production, host exposure, or effects in target tissues. This narrative review summarizes upstream influences, including gastrointestinal physiology, dietary substrates, comorbidities, and medications, and discusses the origins and host processing of major metabolites, their immunometabolic effects, and associated organ phenotypes. Studies in some older populations report lower fecal short-chain fatty acid concentrations. Microbiota-derived indole derivatives and host-synthesized, microbiota-modified bile acids show molecule- and population-specific patterns. Circulating concentrations of some host–microbe co-metabolites are higher in selected older or disease populations, but are also shaped by host biotransformation and clearance. Human evidence is predominantly observational and links metabolic features to health status or clinical phenotypes, whereas animal and cell studies support effects on barrier function, inflammation, and cellular metabolism under specific experimental conditions. Personalized nutrition, metabolite supplementation, selective targeting of microbial enzymes, and multi-omics-based responder stratification are promising research directions; prospective evidence that these strategies improve functional outcomes in older adults remains limited.

## 1 Introduction

Global population aging is accelerating. The World Health Organization projects that the number of people aged 60 years or older worldwide will increase from 1 billion in 2020 to 1.4 billion by 2030 (1). As the older population grows, maintaining organ function and reducing the burden of age-related diseases have become important priorities in aging research.

Intrinsic processes such as genomic instability, telomere attrition, and mitochondrial dysfunction are important foundations of aging. Immune regulation and inter-organ metabolic interactions are also associated with age-related inflammation and changes in organ function. DNA damage can be linked to inflammation through DNA damage responses, cytosolic DNA sensing, and the senescence-associated secretory phenotype (2). Immunosenescence involves remodeling of immune cell composition, effector function, and basal inflammatory status (3). In addition, post-transcriptional regulation mediated by RNA-binding proteins is involved in cellular stress and proteostasis; its dysregulation has been implicated in tissue changes in some aging-related conditions and diseases (4).

Age-related features of the gut microbiota vary with health status and are associated with barrier function, immune regulation, and host metabolism (5). In a Chinese cohort of 1,575 individuals aged 20–117 years, the 297 centenarians had greater microbial species evenness and some microbiota features associated with younger age than younger older-adult controls (6). Animal transfer experiments have shown that microbiota from aged donors can alter intestinal barrier and tissue inflammation-related measures in young mice, whereas transfer of microbiota from young donors can attenuate some inflammatory changes in aged mice (7). In a separate germ-free mouse experiment, transfer of microbiota from aged donors was followed by increased small-intestinal inflammation and systemic T-cell activation; serum immunostimulatory activity associated with bacterial components also increased in recipient mice (8). These studies provide evidence of compositional associations in specific human populations and intervention effects in mouse models, and illustrate why microbiota composition alone cannot establish metabolic function.

This review organizes the evidence according to metabolite origin and host processing. Some metabolites are produced directly by microorganisms, whereas host–microbe co-metabolites arise through metabolic steps involving both partners. For example, trimethylamine (TMA) produced by microbes is oxidized by the host liver to form trimethylamine N-oxide (TMAO) (9). Kynurenine (KYN) is generated mainly through host tryptophan metabolism, although this pathway can be influenced by the microbiota and inflammatory status (10). Accordingly, circulating levels of microbiota-associated metabolites should be interpreted in light of substrate availability, intestinal absorption, host biotransformation, tissue distribution, and hepatic and renal clearance.

Few studies have measured microbial production, metabolic flux, circulating and tissue exposure, and host effects sequentially within the same system. Human evidence is predominantly observational, and longitudinal and interventional validation remains limited. This review discusses upstream factors shaping age-related changes in gut microbial metabolism, major metabolites and their host processing, immunometabolic effects, organ-related phenotypes, and current approaches to modulating microbial metabolic function.

## 2 Materials and methods

This article is a narrative review based primarily on searches of PubMed and Google Scholar, last updated in September 2026. In PubMed, representative keyword combinations covered:

(1) (gut microbiota OR gut microbiome) AND (aging OR aging OR older adults OR longevity OR frailty);
(2) (gut microbiota OR gut microbiome) AND (short-chain fatty acids OR indoles OR bile acids OR trimethylamine N-oxide OR p-cresyl sulfate) AND (aging OR older adults);
(3) (microbial metabolites OR microbiota-derived metabolites) AND (immunometabolism OR inflammation) AND (brain OR liver OR cardiovascular OR skeletal muscle); and
(4) (gut microbiota OR microbial metabolism) AND (dietary intervention OR metabolite supplementation OR microbial enzyme inhibition OR multi-omics prediction) AND (aging OR older adults). Related keywords were adapted for Google Scholar and for the specific metabolites, organ systems, and interventions discussed in each section. Searches were limited to English-language publications.

We included human cross-sectional, longitudinal, and intervention studies relevant to the topic, as well as animal, cell, enzymatic, and computational studies. Reviews and systematic reviews were used for background. We excluded publications unrelated to the topic, conference abstracts, case reports, and articles for which the full text was unavailable. Records were initially screened by title and abstract, followed by full-text assessment. Uncertain records were discussed within the research group. Zotero was used to manage and deduplicate records, followed by manual checking for duplicates.

Original studies were used to support specific findings, whereas reviews were used mainly for background and conceptual context. Multiple reports from the same cohort were selected according to their relevance to the specific point and the outcomes reported. Evidence synthesis considered study design, study population, and actual measurements. Health status and disease context were distinguished, as were metabolite origin, concentration, predicted function, tissue exposure, and metabolic flux. No meta-analysis or formal risk-of-bias assessment was performed.

## 3 Aging-related changes in the gut microbiota and its metabolic functions

### 3.1 Microbiota composition and metabolism-related features across age and health status

Age-related features of the gut microbiota vary with health status. Wilmanski et al. (11) analyzed microbiota composition across multiple populations and found that the positive association between age and microbiome uniqueness at advanced ages was most apparent in relatively healthy individuals and was not evident in those with poorer health. Microbiome uniqueness was also associated with plasma phenylacetylglutamine and several tryptophan metabolites. These findings link community composition with circulating metabolic features; however, circulating concentrations are also affected by host processing and clearance and therefore cannot establish changes in microbial production capacity.

Studies of centenarians have reported findings at different levels. A study in Guangxi, China, found greater microbial species evenness and some community features associated with younger age in centenarians than in younger older-adult controls (6). A study in Japan detected enrichment of some lithocholic acid derivatives in centenarians' feces and, through bacterial isolation experiments, identified an Odoribacteraceae strain capable of producing isoallolithocholic acid. The relative abundance of some butyrate-producing bacteria was lower in this population (12). The former study focused on community composition, whereas the latter combined fecal metabolite measurements with bacterial functional experiments. Because the studies involved different populations and measurements, their findings should be interpreted in light of geographic, dietary, and methodological differences rather than generalized as a uniform microbiota profile of longevity.

Microbiota and metabolic features in people with frailty are also influenced by health status and disease burden. In older adults, Pu et al. (13) found that 18 microbial taxa and 17 plasma metabolites were associated with frailty severity; some microbial associations were replicated in an external population. Because the frailty index included items related to disease and disability, these associations cannot be interpreted as frailty effects independent of comorbidity. Another age- and sex-matched, community-based cross-sectional study found lower fecal relative abundances of *Faecalibacterium* and *Roseburia* and a higher relative abundance of *Akkermansia* in the frail group, alongside poorer nutritional status, greater comorbidity, and more medication use (14). Comparisons across these studies should distinguish microbiota composition, metabolite concentrations, and actual metabolic activity. Circulating concentrations are also affected by host biotransformation and clearance; the reported associations alone do not establish changes in microbial metabolic capacity during physiological aging.

### 3.2 Upstream factors shaping age-related remodeling of microbial metabolism

Age-related features of microbial metabolism are associated not only with age but also with host health status, diet, medication use, and the mucosal immune environment (15, 16). Gastrointestinal transit time and luminal pH are related to substrate retention and the conditions for microbial growth. In a 9-day multi-omics study of 61 healthy adults, gastrointestinal transit and pH were measured using a wireless capsule in 50 participants. These measures were associated with microbiota composition and metabolic features; longer transit time and higher pH were associated with lower levels of some carbohydrate-fermentation metabolites and higher levels of proteolytic metabolites and breath methane (17). This study illustrates how gastrointestinal physiology may help explain variation in microbial metabolism across individuals, although the participants were not specifically an older-adult cohort.

Diet influences the types and amounts of substrates reaching the colon. Changes in the supply of fermentable carbohydrates may alter microbial substrate use; this process is also influenced by substrate structure and microbial interactions (18). As noted above, gastrointestinal transit time and luminal pH are associated with microbiota composition and metabolic features (17). Observational studies in middle-aged and older adults have reported associations between diet quality, microbiota composition, inflammatory markers, and microbial metabolic activity (19). In the NU-AGE dietary intervention study in older adults, diet-related microbiota changes were accompanied by increased predicted functional potential for short-chain fatty acid (SCFA) production and decreased predicted functional potential for p-cresol and secondary bile acid production (20). These findings were based on inferred microbial metabolic profiles and do not directly demonstrate changes in the production flux of the corresponding metabolites.

Multimorbidity and medication use may also affect the interpretation of microbial metabolic features through differences in food intake, gastrointestinal motility, and the intestinal environment (15). Vich Vila et al. (21) analyzed fecal metagenomic data from general-population and gastrointestinal disease cohorts. After accounting for age, sex, body mass index, and polypharmacy, proton pump inhibitors, metformin, antibiotics, and laxatives remained associated with microbial taxonomic features or metabolic pathways. These cohorts were not specifically older-adult cohorts, but the findings indicate that medication exposure and disease context should be considered when comparing microbiota function in older populations; the observed differences should not be attributed to age alone.

The mucosal immune state is associated with the ecological environment in which the microbiota resides. The mucus layer, secretory immunoglobulin A, and antimicrobial factors contribute to mucosal barrier defense (16, 22); age-related changes in intestinal T cells are also associated with barrier integrity and inflammatory regulation (23). In a comparison of naturally aged and young mice, the aged group had a thinner colonic mucus layer, lower expression of some immunoglobulin-, antimicrobial-factor-, and T-cell-related genes, and concurrent changes in microbiota composition (24). This study documents age-related co-occurrence of mucosal and microbiota changes. Together with the human studies described above, these findings provide context for interpreting differences in microbial metabolism across health states. The conceptual framework is shown in Figure 1.

**Figure 1:**
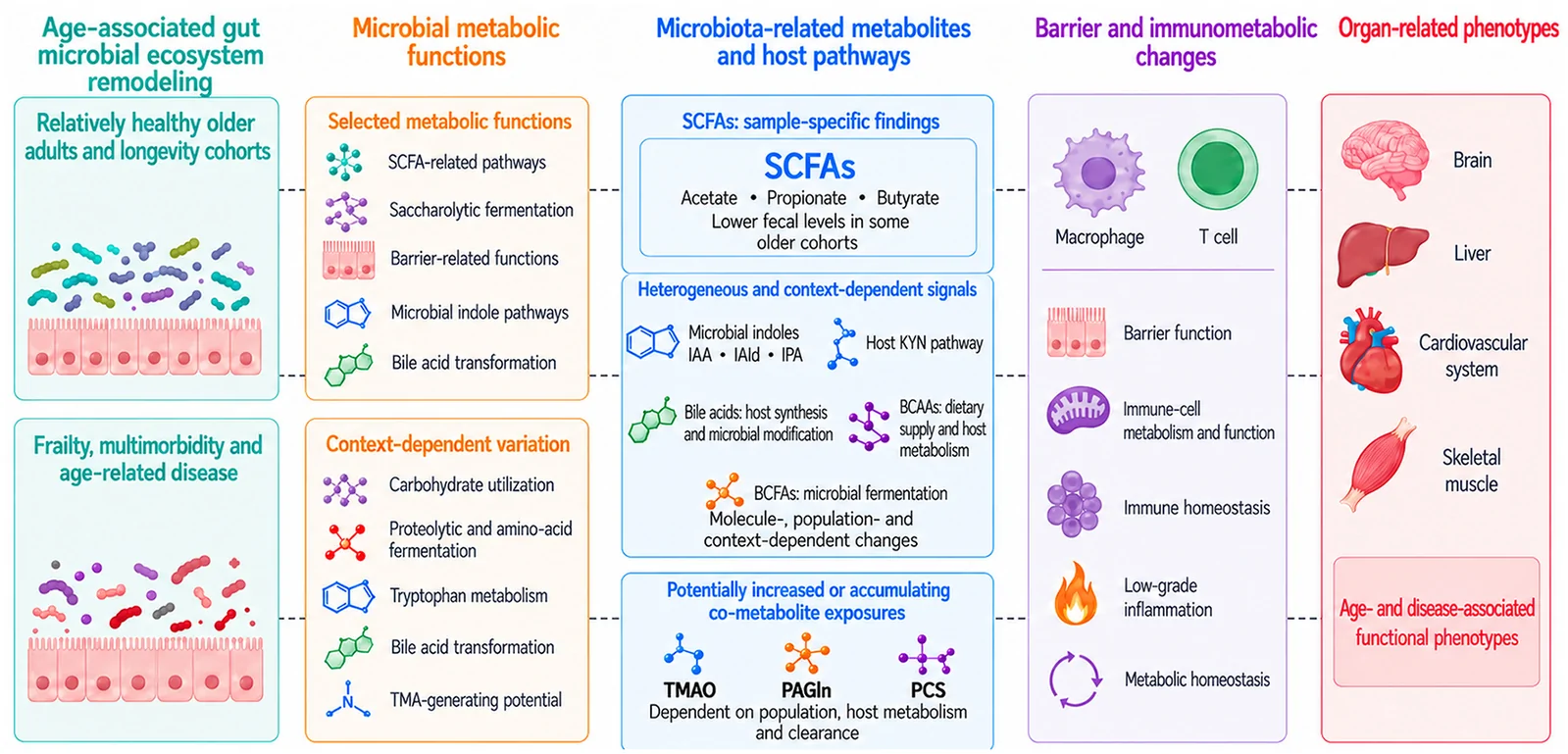
Conceptual framework of gut microbiota-associated metabolism and immunometabolic phenotypes across age and health status. The figure summarizes representative findings on gut microbial ecology, microbial metabolic functions, microbiota-associated metabolites, barrier and immunometabolic changes, and organ-related phenotypes across age, health status, and disease contexts. Relatively healthy older adults, longevity, frailty, multimorbidity, and age-related diseases represent distinct, potentially overlapping research contexts rather than consecutive stages of aging. The metabolites and pathways shown involve microbial production, host–microbe co-metabolism, and processes in which metabolites are produced primarily by the host but are influenced by the microbiota. Dashed lines indicate conceptual links across studies and do not represent a complete causal chain established within a single system. Fecal or circulating metabolite concentrations are not equivalent to microbial production flux; circulating concentrations alone also do not establish target-tissue exposure or effects. The microbiota, barrier, immune-cell, and molecular illustrations are schematic and do not represent microbial compositions, tissue structures, or precise chemical structures shared by specific populations. SCFAs, short-chain fatty acids; TMA, trimethylamine; TMAO, trimethylamine N-oxide; PAGln, phenylacetylglutamine; PCS, p-cresyl sulfate; BCAAs, branched-chain amino acids; BCFAs, branched-chain fatty acids; IAA, indole-3-acetic acid; IAld, indole-3-aldehyde; IPA, indole-3-propionic acid; KYN, kynurenine.

## 4 Microbiota-associated metabolites and age-related immunometabolic changes

### 4.1 Short-chain fatty acids

Short-chain fatty acids (SCFAs), primarily acetate, propionate, and butyrate, are produced by colonic microbial fermentation of dietary carbohydrates and serve as both energy substrates and signaling molecules (25). Human studies have compared age-related differences in SCFAs using different measurement approaches. A meta-analysis of 18 studies found lower fecal concentrations of acetate, propionate, and butyrate in relatively healthy older adults than in younger adults (26). Another observational study used stable-isotope pulse tracing and model-based estimates and found lower whole-body SCFA production rates and interpool fluxes in older adults (27). The former measured fecal concentrations, whereas the latter assessed whole-body metabolic kinetics; whole-body production rates cannot be equated directly with colonic microbial production flux. Dietary substrates, intestinal absorption and transit, and host utilization should also be considered when interpreting these group differences. Sample processing and analytical methods may affect comparisons across studies.

The effects of SCFAs on intestinal homeostasis are first evident in epithelial metabolism. Mature colonic epithelial cells can take up and oxidize butyrate for energy; this process increases epithelial oxygen consumption and helps maintain physiological hypoxia (25). In an antibiotic-treated mouse model, butyrate derived from the class Clostridia maintained epithelial hypoxia through epithelial peroxisome proliferator-activated receptor gamma signaling. Butyrate depletion was accompanied by increased epithelial oxygenation and reduced colonization resistance against fungi (28). This experiment supports a functional relationship among butyrate, the epithelial oxygen environment, and colonization resistance, providing a mechanistic lead for studying age-related changes in the intestinal barrier.

SCFAs can also signal through free fatty acid receptor 2 (FFAR2), free fatty acid receptor 3, and hydroxycarboxylic acid receptor 2 (HCAR2); butyrate and propionate can also inhibit histone deacetylases (25). These actions involve distinct aspects of mucosal immunity. In mice, SCFAs regulated colonic regulatory T cells (Tregs) and interleukin-10 (IL-10) expression through FFAR2 (29). Butyrate-related HCAR2 signaling altered the immunoregulatory activity of colonic macrophages and dendritic cells and enhanced their capacity to induce Tregs (30). In mouse and cell experiments, butyrate also increased histone H3 acetylation at the *Foxp3* locus and promoted colonic Treg differentiation (31). Together, these findings support receptor-mediated and epigenetic mechanisms.

Differences in SCFA measurements between human age groups, considered alongside these experimental mechanisms, suggest that changes in microbial metabolism may be related to age-associated epithelial and mucosal immune states. However, the human comparisons and experimental findings come from different settings; they do not establish that lower fecal SCFA concentrations in older adults cause impaired barrier or immune function. Assessing this relationship will require measurements of local metabolite exposure and functional outcomes in the relevant populations.

### 4.2 Microbial indoles and host kynurenine-pathway metabolites

Tryptophan can be converted by gut microbes into various indole derivatives or enter the kynurenine pathway through host indoleamine 2,3-dioxygenase 1 (IDO1) and tryptophan 2,3-dioxygenase (TDO) (32); the pathways are shown in Figure 2. Human studies have primarily provided age-related observational findings on the host kynurenine pathway. A systematic review found that blood tryptophan (TRP) levels were negatively associated with age, whereas kynurenine (KYN) and the KYN/TRP ratio were generally positively associated with age. However, results varied across studies, most of which used cross-sectional designs (33). A study in people with frailty found that quinolinic acid (QUIN) and the QUIN/KYN ratio were associated with higher interleukin-6 levels, a greater likelihood of frailty, and poorer physical function (34). Another study of 73 older adults found higher serum KYN levels in participants with frailty than in robust or prefrail participants (35). Blood KYN concentrations and the KYN/TRP ratio are also influenced by substrate levels, host metabolism, and clearance; they do not directly measure microbial tryptophan metabolic flux or the activity of a single enzyme. Microbial indole metabolites have not shown a consistent age-related pattern. In a study of a longevity population, fecal indole-3-acetic acid (IAA) and some other indole metabolites were higher in centenarians than in younger older adults (36). These comparisons reflect metabolic features in a specific longevity context.

**Figure 2:**
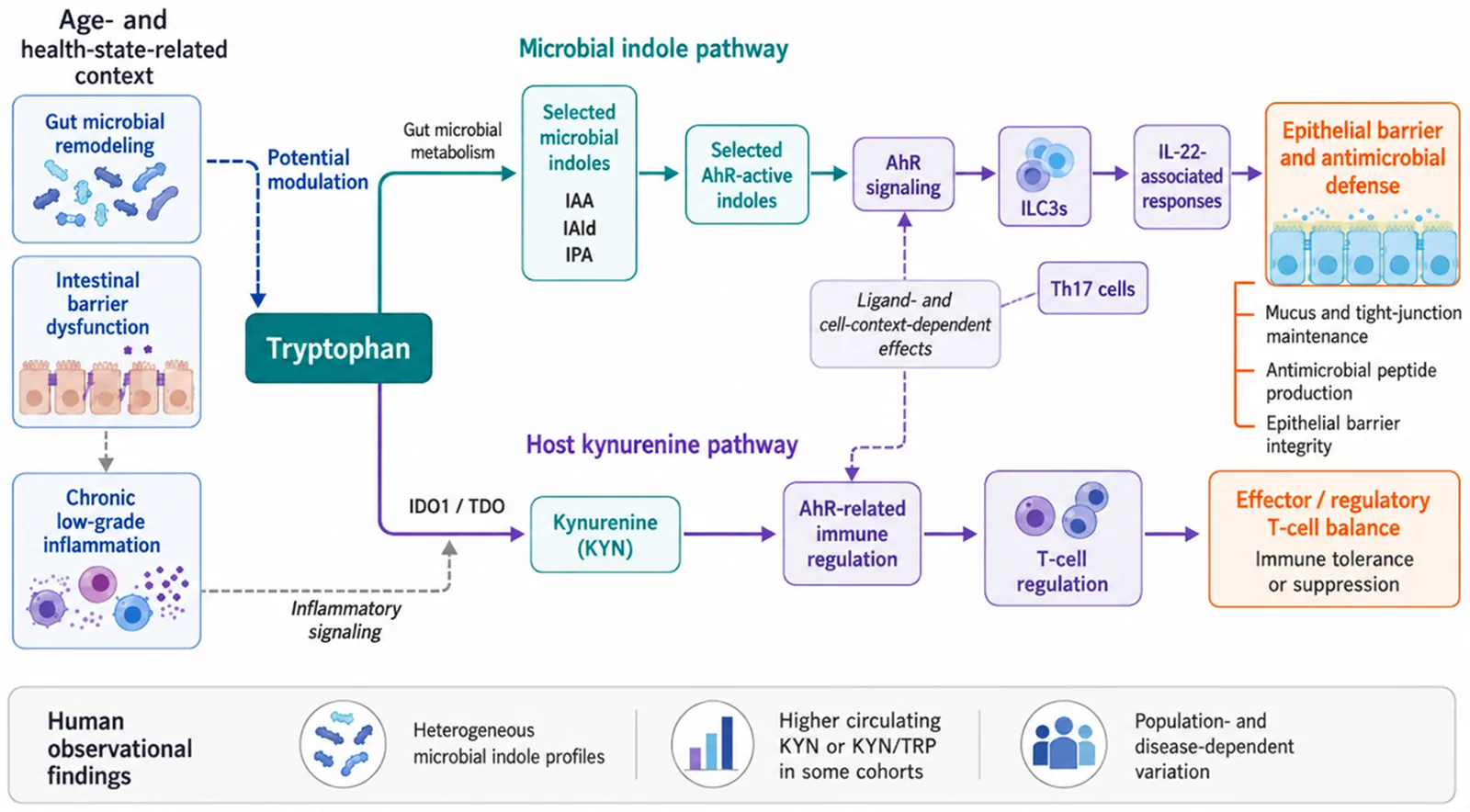
Microbial and host tryptophan metabolic pathways in aging-related studies. Tryptophan can be converted by gut microbes into indole derivatives or enter the kynurenine pathway through host IDO1 and TDO. Some indole derivatives and kynurenine are involved in AhR-related immune regulation, with cellular effects that depend on the ligand and cellular environment. The illustrated IAld–AhR–ILC3–IL-22 pathway does not indicate that Th17 cells participate in this specific pathway. The background factors on the left are not shared features of all physiologically aging populations and do not represent an inevitable sequence of events. Solid lines summarize steps supported under the corresponding experimental conditions; dashed lines indicate potential regulatory or indirect links. The figure does not imply that the entire pathway has been validated in human physiological aging. Current evidence does not establish a general shift in tryptophan metabolic flux from microbial indole production toward kynurenine metabolism. Circulating KYN and the KYN/TRP ratio do not directly represent microbial metabolic flux or the activity of a single enzyme; their interpretation should consider host metabolism and clearance. IAA, indole-3-acetic acid; IAld, indole-3-aldehyde; IPA, indole-3-propionic acid; KYN, kynurenine; TRP, tryptophan; IDO1, indoleamine 2,3-dioxygenase 1; TDO, tryptophan 2,3-dioxygenase; AhR, aryl hydrocarbon receptor; ILC3s, group 3 innate lymphoid cells; Th17, T helper 17 cells; IL-22, interleukin-22.

Some microbial indole metabolites can participate in mucosal immune regulation through the aryl hydrocarbon receptor (AhR), with effects depending on the ligand and cellular environment (37, 38). In a mouse experiment, *Lactobacillus reuteri* converted tryptophan to indole-3-aldehyde (IAld), which promoted interleukin-22 production by group 3 innate lymphoid cells through AhR and enhanced mucosal defense in the experimental model (39). Another study involving a longevity population identified a Clostridium scindens strain capable of producing IAA. Administration of the strain or IAA improved intestinal barrier-related phenotypes in aged mice, with results involving intestinal AhR signaling (36). The first study examined the mucosal immune effects of a specific indole, whereas the second addressed human microbiota and metabolite features, bacterial production capacity, and intervention in aged mice as distinct levels of evidence.

The host kynurenine pathway is also involved in immune regulation. In stimulation experiments using human peripheral-blood conventional dendritic cells, IDO1 expression and KYN production increased. Under conditions in which the cell supernatant inhibited T-cell proliferation, IDO1 blockade partially restored proliferation (40). Another experiment showed that KYN activated AhR and promoted the generation of Foxp3-positive Tregs in a mouse T-cell system (41). These studies support specific links among IDO1, KYN, and immune-cell responses under the conditions examined. Together with the human findings described above, the evidence addresses associations between metabolic measures and age or health status, as well as immunoregulatory mechanisms in specific experimental models. Although microbial indoles and host-derived KYN can both involve AhR, their origins, experimental contexts, and target cells should be considered separately.

### 4.3 Bile acids

Bile acids (BAs) are synthesized by the host and can be further modified by gut microbes. The liver produces primary BAs, including cholic acid (CA) and chenodeoxycholic acid (CDCA). After entering the gut, they can be converted by microbial deconjugation, 7α-dehydroxylation, and redox reactions into deoxycholic acid (DCA), lithocholic acid (LCA), and their derivatives (42, 43). Enzymatic mechanisms have been established for specific microbial BA transformations: Funabashi et al. identified the complete enzymatic pathway for conversion of CA to DCA (44). Thus, BA pool composition reflects both host synthesis and microbial transformation, as well as subsequent host processing (45).

Human studies have not shown that all BAs change in the same direction with age. A cross-sectional study of 100 relatively healthy older adults and 100 younger adults found no marked difference in total fecal or serum BA levels between groups. The older group had lower fecal CA, CDCA, and primary-to-secondary BA ratios, but higher LCA in fecal and serum samples (46). The study excluded participants with diagnosed chronic diseases and those who had recently used certain medications affecting the microbiota or BA metabolism. However, some liver- and kidney-function-related measures differed between the older and younger groups and should be considered when interpreting serum results. A study of Japanese centenarians found enrichment of several LCA isomers in feces, including 3-oxolithocholic acid (3-oxoLCA) and isoallolithocholic acid (isoalloLCA). The researchers also identified an Odoribacteraceae strain and associated enzymes capable of producing isoalloLCA from centenarian fecal samples (12). These studies describe BA profiles in a relatively healthy older-adult group and a specific longevity population, respectively; their findings do not establish a continuous aging trajectory.Differences in fecal or serum concentrations also do not directly quantify microbial transformation flux.

Structural differences among specific BAs can affect immune-cell differentiation under experimental conditions. Mouse and cell experiments showed that 3-oxoLCA bound to retinoic acid receptor-related orphan receptor gamma t and inhibited T helper 17 (Th17) cell differentiation. In contrast, isoalloLCA promoted regulatory T-cell (Treg) differentiation through mitochondrial reactive oxygen species and Foxp3 conserved non-coding sequence 3 (47). Although both are LCA derivatives, they act through different pathways, and their immune effects cannot be inferred from total BA levels.

BAs can also regulate immune cells through receptor signaling. Farnesoid X receptor (FXR) is a nuclear receptor, whereas G protein-coupled bile acid receptor 1 (TGR5) is a membrane receptor. CDCA is an important endogenous ligand of FXR (48). LCA, DCA, and other BAs can activate TGR5 (48, 49). In a mouse model of chemical colitis, TGR5 deficiency was accompanied by increased recruitment of proinflammatory macrophages, while receptor agonism altered macrophage phenotype through an IL-10-related mechanism (50). This finding supports a receptor role in the inflammatory model studied and should not be combined directly with BA profile differences in ordinary older adults as though they reflected the same mechanism.

Other experiments further illustrate differences in target cells and tissue contexts. In dendritic-cell-related experiments, isodeoxycholic acid (isoDCA) reduced immunostimulatory activity and promoted Foxp3 induction. Dendritic-cell FXR deficiency produced some similar transcriptional and Treg-cell effects, but phenotypic similarity alone does not establish FXR as the sole target through which isoDCA acts (51). In naturally aged mice, brain levels of tauro-β-muricholic acid (TβMCA) were elevated. *In vitro* and animal experiments separately examined microglial inflammatory responses and neurobehavioral changes, while metagenomic findings linked its accumulation to a reduction in bacteria carrying bile salt hydrolase (52). Another study found that LCA binds to Tubby-like protein 3 (TULP3) and regulates muscle function in aged animals through a sirtuin–vacuolar ATPase–AMP-activated protein kinase (AMPK) signaling pathway (53). These studies concern different molecules, tissues, and experimental conditions and support distinct mechanistic steps; they cannot yet be combined into a unified BA pathway validated in human physiological aging.

### 4.4 Trimethylamine N-Oxide and p-Cresyl sulfate

Gut microbes can convert dietary substrates such as choline and L-carnitine into trimethylamine (TMA), which is then oxidized by the host to trimethylamine N-oxide (TMAO). Gut microbes can also convert tyrosine to p-cresol, which is subsequently sulfated by the host to form p-cresyl sulfate (PCS) (54). TMAO and PCS are both host–microbe co-metabolites, but their microbial precursors and host transformations differ.

TMAO is formed through sequential microbial and host metabolism. Gut microbes can convert substrates such as choline and L-carnitine to TMA through different pathways. After absorption, TMA is oxidized mainly by hepatic flavin-containing monooxygenase 3 to TMAO (55). In a cross-sectional study of 451 patients aged 65 years or older with cardiovascular disease, plasma TMAO concentrations were higher in participants with frailty than in those without frailty; higher concentrations were also associated with physical and cognitive frailty (56). This association arose in the context of cardiovascular disease, and participants with frailty also differed from those without frailty in kidney function and disease burden. Plasma concentrations therefore cannot, by themselves, reflect microbial TMA production. In a separate vascular-inflammation experiment, TMAO treatment of human umbilical vein endothelial cells reduced SIRT3 expression and SOD2 activity, increased mitochondrial reactive oxygen species, and was accompanied by NLRP3 inflammasome activation. Inhibiting NLRP3 or interfering with SIRT3-related signaling attenuated some inflammatory responses. Corresponding changes were also observed in the aortas of choline-fed ApoE–/– mice (57). These experiments identified steps involved in vascular inflammation under the specified treatment conditions; their context differs from human physiological aging.

PCS arises from microbial tyrosine metabolism followed by host sulfation: gut microbes produce p-cresol, which is absorbed and converted to PCS (58, 59). A cross-sectional study of 180 healthy participants aged 20–90 years found higher serum PCS concentrations in the group aged 65 years or older. PCS was positively associated with age but was not associated with serum tyrosine concentrations (60). This finding describes differences between age groups rather than longitudinal change within individuals. PCS is highly protein-bound, and its circulating levels are also affected by host sulfation and renal clearance (59). Therefore, the serum concentration difference in this study cannot be attributed solely to increased microbial conversion of tyrosine.

### 4.5 Branched-chain amino acids and branched-chain fatty acids

Branched-chain amino acids (BCAAs) comprise leucine, isoleucine, and valine. They derive mainly from dietary intake and host protein metabolism, but can also serve as substrates for gut microbial fermentation. Specific microbial taxa can convert them into branched-chain fatty acids (BCFAs), including isovalerate, 2-methylbutyrate, and isobutyrate. Circulating BCAA concentrations are jointly influenced by intake, absorption, host tissue metabolism, and microbial utilization; fecal or circulating BCFA concentrations also do not directly represent microbial production flux. In ApoE–/– mice fed a high-fat diet, Qiao et al. (61) found that the porA gene of Parabacteroides merdae was involved in BCAA degradation. Administration of this bacterium enhanced intestinal BCAA degradation and reduced atherosclerotic lesions, whereas deletion of porA attenuated these effects. Plaque mTORC1 signaling also changed following the intervention. This study supports the involvement of *porA*-related metabolic function in the bacterial effect in this disease model; the mediating role of the mTORC1 change requires targeted testing.

Associations between circulating BCAAs and health status have not been consistent in direction. The Seniors-ENRICA 2 cohort followed 859 community-dwelling adults aged 65 years or older for 5 years; lower plasma isoleucine, leucine, and valine levels were associated with the study-defined healthy-aging outcome (62). A cross-sectional study in a longevity region of Guangxi included 1,034 residents aged 20–110 years. Among the 597 adults aged 86–110 years who completed frailty assessments, higher serum BCAA concentrations were associated with lower frailty scores; this association remained observable after adjustment for age and body mass index (63). These studies examined follow-up outcomes in community-dwelling older adults and concurrent frailty in very old adults from a specific region, respectively. Protein intake, nutritional status, and muscle metabolism may influence circulating BCAA levels, but the available findings cannot distinguish the contributions of these processes from microbial utilization or establish a uniform age-related direction of change in BCAAs.

Experimental studies of microbiota-derived metabolites provide another type of evidence. Wang et al. (64) administered *P. merdae* or three BCFAs to D-galactose-induced accelerated-aging mice. Some intervention groups showed lower frailty index scores and reduced interleukin-6 and tumor necrosis factor-α levels in colonic tissue homogenates, accompanied by improvements in some oxidative stress and glucose metabolism measures. These findings support bacterial and metabolite intervention effects in the models studied; whether the effects of the bacterial intervention were mediated by the measured BCFAs requires testing within the same experiment. Human studies report associations between circulating BCAAs and health status, whereas animal studies examine effects in atherosclerosis or accelerated-aging models; these contexts should be interpreted separately. The origins, representative effects, and scope of evidence for BCAAs and their microbial metabolites are summarized in Table 1.

**Table 1:** Major microbiota-associated metabolites in studies of aging and related diseases.

Metabolite class	Source and host processing	Aging- or disease-related findings	Experimentally supported mechanisms	Evidence type and scope
Short-chain fatty acids (SCFAs)	Produced by colonic microbial fermentation of dietary carbohydrates; subsequently absorbed and used by the host (25).	A meta-analysis found lower fecal acetate, propionate, and butyrate concentrations in older than younger adults (26). A human tracer study estimated lower whole-body SCFA production rates and interpool fluxes in older adults (27).	Mouse and cell experiments support butyrate-associated colonic regulatory T-cell differentiation and increased histone acetylation at the *Foxp3* locus (31).	Human age-group comparisons and tracer-based kinetic estimates; experimental effects are limited to the corresponding models.
Microbial indole derivatives	Gut microbes convert tryptophan into indole derivatives; some products may undergo further host processing (32).	In a specific centenarian population, fecal indole-3-acetic acid (IAA) and some other indole metabolites were higher (36).	In mice, indole-3-aldehyde (IAld) participates in aryl hydrocarbon receptor (AhR)-related interleukin-22 (IL-22) mucosal responses (39).	Observational findings in a longevity population; experimental effects are limited to the studied molecules and models.
Host kynurenine-pathway metabolites	Tryptophan primarily enters the kynurenine pathway through host indoleamine 2,3-dioxygenase 1 (IDO1) and tryptophan 2,3-dioxygenase (TDO) (32).	A systematic review found that blood kynurenine (KYN) concentrations and KYN-to-tryptophan (TRP) ratios were generally positively associated with age, with heterogeneity across studies (33).	Mouse T-cell experiments support KYN–AhR signaling in regulatory T-cell (Treg) generation (41).	Human studies support age-related associations; KYN concentrations and KYN/TRP ratios are not direct measures of microbial flux or single-enzyme activity.
Bile acids (BAs)	Primary BAs are synthesized by the host and further modified by gut microbes (42, 43).	In relatively healthy older adults, fecal cholic acid (CA) and chenodeoxycholic acid (CDCA) were lower, whereas fecal and serum lithocholic acid (LCA) were higher (46). Several LCA derivatives, including 3-oxolithocholic acid (3-oxoLCA) and isoallolithocholic acid (isoalloLCA), were enriched in feces from a specific centenarian population (12).	Mouse and cell experiments found that 3-oxoLCA inhibited T helper 17 (Th17) cell differentiation, whereas isoalloLCA promoted Treg differentiation (47).	Human findings vary by metabolite and population; mechanistic findings are limited to specific BA derivatives and experimental conditions.
Trimethylamine N-oxide (TMAO)	Microbes produce trimethylamine (TMA), which is then oxidized by the host liver to TMAO; circulating levels are also affected by host processing (54, 55).	In older adults with cardiovascular disease, higher plasma TMAO was associated with frailty (56).	Endothelial-cell and mouse experiments support TMAO-related effects involving sirtuin 3 (SIRT3), superoxide dismutase 2 (SOD2), oxidative stress, and NLRP3 inflammasome activation (57).	Cross-sectional association in a disease population; experimental findings are limited to the corresponding treatment conditions.
p-Cresyl sulfate (PCS)	Microbes produce p-cresol, which is sulfated by the host; circulating levels are affected by protein binding and renal clearance (58, 59).	A cross-sectional study of healthy participants found a positive association between serum PCS and age (60).	The cited studies did not test a mechanism directly linking PCS to an aging phenotype.	Human age-related association; serum concentrations do not distinguish precursor formation, host conversion, and clearance.
Branched-chain amino acids (BCAAs)	Derived primarily from dietary intake and host protein metabolism; certain microbes can use or degrade them (61, 64).	In a longitudinal study of community-dwelling older adults, lower plasma BCAAs were associated with healthy aging (62). In a cross-sectional study of older adults in a longevity region, higher serum BCAAs were associated with lower frailty scores (63).	In high-fat-diet apolipoprotein E knockout (ApoE–/–) mice, *Parabacteroides merdae* porA-related BCAA degradation was associated with plaque phenotypes (61).	Human findings concern different populations and outcomes; experimental evidence comes from an atherosclerosis model.
Branched-chain fatty acids (BCFAs)	Can be produced through microbial fermentation of BCAAs; measured concentrations are also affected by absorption and downstream metabolism (64).	In D-galactose-induced mice, *P. merdae* or selected BCFA interventions lowered the frailty index and colonic tissue inflammatory markers in some groups (64).	Animal interventions support phenotypic effects in the model; a direct host target has not been established (64).	Evidence from an accelerated-aging model; not evidence of effects in human physiological aging.

This table provides a descriptive summary of evidence types and scope; no formal evidence-quality grading was performed. Experimental effects are limited to the corresponding models and exposure conditions. Fecal or circulating concentrations are not direct measures of microbial production flux, and whole-body production rates are not equivalent to colonic microbial production flux.

## 5 Microbial metabolic networks and aging-related chronic diseases

### 5.1 The gut–brain immunometabolic axis in cognitive aging and neurodegeneration

Experimental inflammation models have shown effects of microbiota-associated metabolites on brain endothelial cells and microglia. In an *in vitro* blood–brain barrier (BBB) model, physiologically relevant concentrations of butyrate and propionate altered actin organization and tight-junction protein localization in brain endothelial cells, attenuating lipopolysaccharide-induced impairment of barrier function and mitochondrial networks (65). In adult mice, inulin intake reduced the inflammatory response of isolated microglia to lipopolysaccharide; direct treatment of primary microglia with acetate or butyrate also reduced cytokine release (66). In another study, exogenous trimethylamine N-oxide (TMAO) was administered to senescence-accelerated mouse prone 8 (SAMP8) mice and age-matched senescence-accelerated mouse resistant 1 (SAMR1) mice. Neuronal senescence, mitochondrial damage, and oxidative stress increased, while the expression of synaptic-plasticity-related proteins decreased, accompanied by impaired learning and memory (67). These experiments separately support barrier and microglial responses under inflammatory conditions and TMAO intervention effects in a specific mouse model.

Alzheimer's disease (AD) studies include two distinct types of evidence: human bile acid profiles and indole interventions in animals. A baseline cross-sectional analysis of 1,464 cognitively normal participants and participants with mild cognitive impairment or AD found lower serum cholic acid and higher deoxycholic acid and some of its conjugates in participants with AD. Some secondary-to-primary bile acid ratios were associated with poorer cognitive performance; this relationship was also supported in independent serum and brain-tissue samples (68). These ratios reflect differences in metabolic profiles and do not directly measure microbial conversion flux. In another study, treatment of APP/PS1 mice with indole, indole-3-acetic acid, and indole-3-propionic acid reduced neuroinflammation, amyloid deposition, and Tau hyperphosphorylation, and improved synaptic and cognitive phenotypes (69). The studies concern distinct metabolic pathways: the human findings are disease-related associations, whereas the animal findings support intervention effects within a model.

Parkinson's disease (PD) studies also require gastrointestinal function to be considered when interpreting metabolite concentrations. In patients with PD, a cross-sectional study found lower fecal short-chain fatty acid concentrations but higher plasma concentrations of some short-chain fatty acids; both lower fecal butyrate and higher plasma butyrate were associated with poorer cognitive performance (70). Another cross-sectional study of 250 patients with PD and 250 controls found higher plasma phenylacetylglutamine (PAGln), p-cresol conjugates, and indoxyl sulfate in patients. After adjustment for age, sex, and medication use, PAGln and p-cresyl glucuronide remained associated with motor symptom severity, with no corresponding association with cognitive function (71). These co-metabolites were also associated with constipation; fecal and circulating concentrations should not be interpreted directly as measures of microbial production. In α-synuclein-overexpressing mice, a high-fiber prebiotic diet reduced nigral protein aggregation, altered microglial states, and improved motor performance. Depletion of microglia using a colony-stimulating factor 1 receptor (CSF1R) inhibitor abolished the motor benefit, supporting microglial involvement in the dietary intervention effect (72).

Human studies provide associations between metabolic features and clinical phenotypes in AD and PD, whereas experimental studies support barrier, neuroimmune, and neurological effects under specific conditions. Direct evidence relevant to physiological cognitive aging remains limited. Future longitudinal studies should combine metabolite-exposure measurements with targeted interventions to determine whether these changes precede changes in cognitive function.

### 5.2 The gut–liver immunometabolic axis in aging and metabolic liver disease

The liver receives gut-derived signals through the portal vein, synthesizes bile acids, and participates in the transformation of host–microbe co-metabolites. Its immunometabolic responses involve hepatocytes, Kupffer cells, and other cell populations (73). In studies of metabolic dysfunction-associated steatotic liver disease (MASLD), senescent hepatocytes have been associated with altered liver function. To examine paracrine effects *in vitro*, researchers induced senescence in Huh7 cells and treated other cells with the resulting conditioned medium. Recipient Huh7 cells developed secondary senescence, while inflammation-related markers in macrophages and profibrotic markers in hepatic stellate cells increased (74). These findings describe intercellular effects in a disease-research context and do not involve intervention with gut-derived metabolites.

Bile acid composition in naturally aged mice varies by sex and tissue. A comparison of 3- and 26-month-old mice found a higher serum primary-to-secondary bile acid ratio in aged mice, along with changes in bile acid profiles in the liver and intestine. Deoxycholic acid increased in the liver and colon of males but decreased in females. Co-housing with young mice altered the microbiota composition and some bile acid measures in aged mice (75). Another study involving seven days of bile acid feeding in mice and exposure of primary cells found that lithocholic acid mainly induced inflammatory responses in Kupffer cells, whereas deoxycholic acid promoted an early profibrotic response in hepatic stellate cells. The interleukin-1β response induced by deoxycholic acid plus ATP was reduced in NLRP3-deficient cells (76). The former study measured bile acid profiles under aging and co-housing conditions; the latter examined cellular responses to exposure to specific bile acids.

In patients with biopsy-confirmed metabolic dysfunction-associated fatty liver disease (MAFLD), fecal 3-succinylated cholic acid (3-sucCA) levels were inversely associated with liver injury markers (77). Strain-screening and enzymatic experiments identified a Bacteroides uniformis strain capable of producing 3-sucCA and the associated biosynthetic enzyme BAS-suc. In mice with experimental metabolic dysfunction-associated steatohepatitis (MASH), 3-sucCA was largely confined to the intestinal lumen. Administration of 3-sucCA increased *Akkermansia muciniphila* and improved liver pathology (77). The human fecal association, bacterial biosynthesis experiments, and disease-model intervention concern different study populations and should not be combined into a gut–liver process established in natural aging.

Whether actual hepatic exposure to specific bile acids during natural aging reaches levels that elicit the cellular responses observed experimentally remains to be tested through tissue-exposure measurements and targeted interventions.

### 5.3 The gut–cardiovascular axis in vascular aging and atherosclerosis

Associations between the gut microbiota and age-related differences in endothelial function are supported by human comparisons and animal interventions. After antibiotic pretreatment, young mice receiving microbiota from aged donors showed impaired endothelium-dependent relaxation in the aorta and mesenteric arteries, accompanied by increased vascular oxidative stress and inflammation and altered telomere function (78). This transplantation experiment did not identify a specific metabolic mediator. In adults without apparent clinical disease, a cross-sectional study found higher plasma TMAO in the middle-aged and older groups, with higher levels associated with lower brachial artery flow-mediated dilation. In the same study, TMAO supplementation induced endothelial dysfunction in young mice, whereas inhibition of microbial trimethylamine production in aged mice lowered circulating TMAO and improved vascular oxidative stress and endothelium-dependent relaxation (79). These animal experiments involved interventions targeting the overall microbiota and a specific metabolic pathway, respectively; the transplantation effect cannot therefore be attributed to TMAO.

Studies of atherosclerosis and cardiovascular risk involve different populations and outcomes. In the Guangzhou Nutrition and Health Study, multi-omics samples collected midway through follow-up showed that microbiota features and fecal metabolites statistically explained some variation in the serum metabolome. A score based on 32 associated serum metabolites was associated with the occurrence of metabolic syndrome and carotid plaques (80). The temporal relationship between sampling and each outcome should be evaluated for the specific analysis. Another study found lower circulating indole-3-propionic acid (IPA) in patients with coronary artery disease. IPA supplementation reduced plaque formation in apolipoprotein E-deficient mice and promoted cholesterol efflux to apolipoprotein A-I in mouse and human macrophages; an animal experiment also found increased macrophage reverse cholesterol transport (81). PAGln is formed when microbially produced phenylacetic acid undergoes host conjugation (82). A study in a population undergoing cardiovascular assessment found that plasma PAGln was associated with subsequent major adverse cardiovascular events (83). These human findings concern plaque, coronary artery disease, and event risk, respectively, and should not be combined into a shared metabolic trajectory of physiological vascular aging.

Platelet responses and thrombosis represent another category of outcomes. *Ex vivo* experiments with human platelets showed that TMAO enhanced platelet responses to agonists. Dietary and microbiota interventions in mice supported an effect of TMAO-related pathways on experimental thrombosis. In a clinical cohort undergoing elective coronary angiography, circulating TMAO was associated with subsequent cardiovascular event risk (84). Other platelet and mouse experiments showed that PAGln enhanced platelet responses and experimental thrombosis through signaling involving α2A-, α2B-, and β2-adrenergic receptors; receptor intervention attenuated these effects (83). TMAO and PAGln are both host–microbe co-metabolites, and their circulating concentrations are also influenced by substrate supply, host transformation, and clearance. Current evidence separately supports associations with age-related endothelial function, risk associations in disease cohorts, and thrombosis mechanisms in experimental models. The next step is to examine the temporal relationship between changes in metabolite exposure and vascular function in longitudinal cohorts with clearly characterized health status.

### 5.4 The gut–muscle axis in muscle aging and sarcopenia

Human observational studies show associations between gut microbiota features and muscle mass, muscle strength, and physical function, but no consistent microbiota profile of sarcopenia has emerged. A systematic review of 32 studies involving 10,781 participants found lower microbial richness and diversity in some populations with sarcopenia or low muscle mass, while associations with specific taxa varied in direction. The mean ages in the included populations ranged from 50 to 85 years, and the settings included community, hospital, and long-term care populations. Differences in sarcopenia definitions, nutritional and medication backgrounds, and microbiota assessment methods also affect comparisons (85). In an experiment in naturally aged animals, 88-week-old female rats receiving fecal microbiota transplantation from young donors for 8 weeks had greater muscle mass, grip strength, and exercise capacity than rats receiving microbiota from aged donors. These changes were accompanied by increases in muscle fiber cross-sectional area, satellite cell number, and mitochondria-related measures (86). The transplantation regimen altered muscle phenotypes in aged rats; concurrent changes in serum metabolites have not been established as mediators.

Evidence from a specific metabolite intervention comes from a different model. In SAMP8 mice, a model of accelerated aging, combined acetate, propionate, and butyrate supplementation in drinking water for 3 months beginning at 7 months of age increased muscle mass, muscle fiber cross-sectional area, grip strength, and *ex vivo* contractile force and improved fatigue resistance. In muscle tissue, AKT/mTOR signaling, PGC1α protein, and mitochondrial DNA-related measures increased, while the expression of muscle atrophy-related genes decreased. In C2C12 myotubes, short-chain fatty acids increased myotube diameter; rapamycin attenuated this effect, supporting the involvement of mTOR signaling in this cellular response (87). Tissue molecular measures reflect changes in related signaling; these supplementation experiments and the fecal microbiota transplantation experiment addressed metabolite exposure and overall microbiota intervention, respectively.

Findings in patients with chronic kidney disease illustrate the importance of host clearance when interpreting metabolite associations. A *post-hoc* analysis of baseline data from the RECOVERY study found that nondialysis patients with higher serum total indoxyl sulfate had lower skeletal muscle mass index and grip strength. Indoxyl sulfate remained associated with sarcopenia in a model that included age, sex, diabetes, creatinine, and the ratio of myostatin to skeletal muscle mass index (88). Serum indoxyl sulfate was also inversely associated with estimated glomerular filtration rate; this cross-sectional association in a disease population should therefore be interpreted in light of renal clearance. Current studies provide human microbiota associations, microbiota-intervention effects in aged animals, short-chain fatty acid effects in an accelerated-aging model, and co-metabolite associations in people with chronic kidney disease. Future studies should test whether changes in exposure to specific metabolites mediate muscle-function benefits following microbiota interventions. Associations and experimental mechanisms involving these organs are shown in Figure 3.

**Figure 3:**
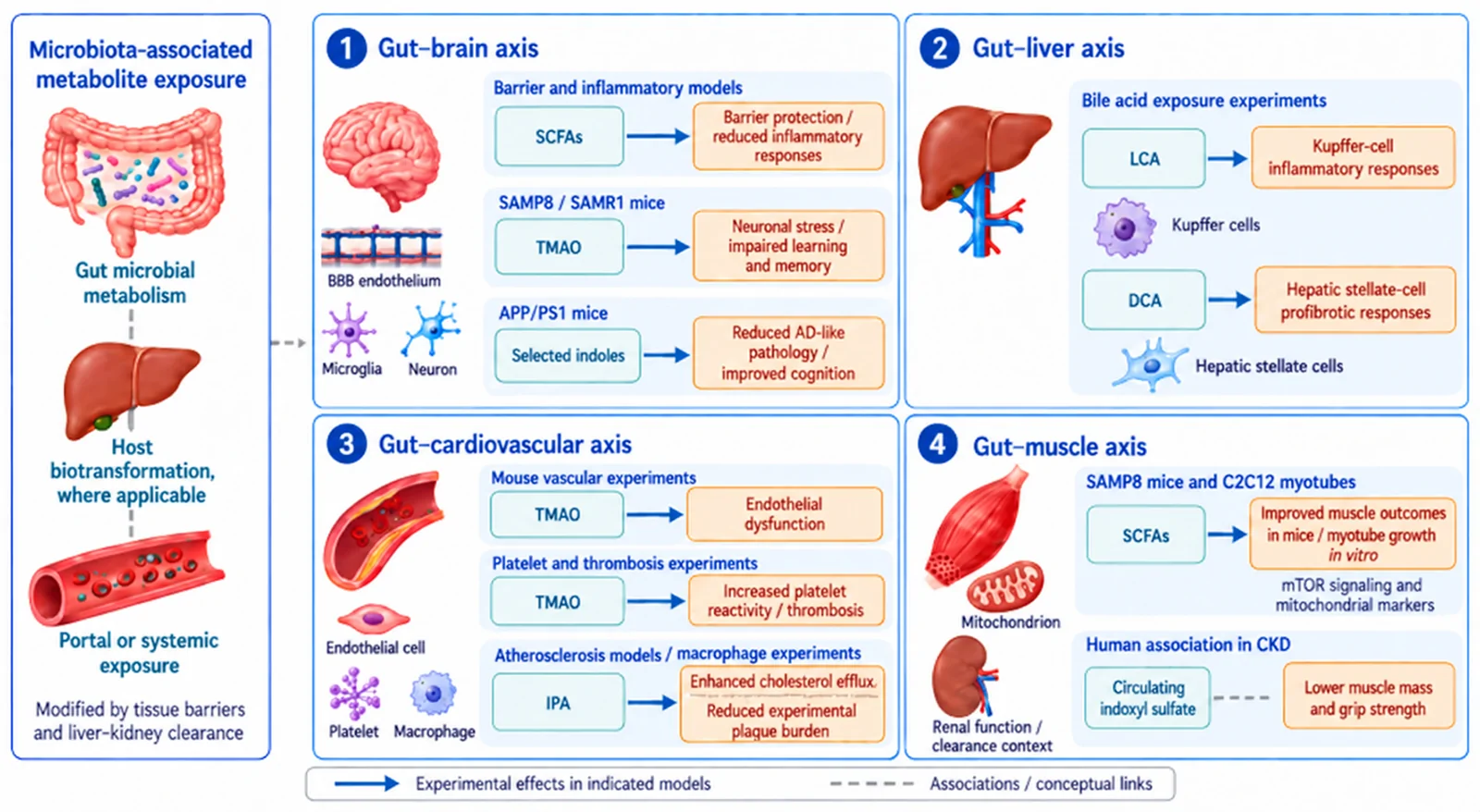
Representative associations and experimental effects of microbiota-associated metabolites on multi-organ phenotypes. The panels show representative findings from experimental barrier and inflammation studies, aging-related animal models, and age-related disease studies; these should not all be interpreted as evidence of physiological aging. Solid arrows indicate effects or intervention responses supported under the corresponding experimental conditions. Dashed lines without arrowheads indicate observational associations or conceptual links, with the specific meaning depending on the connected nodes. Results shown together do not establish mediation between them. The framework on the left depicts the production, host processing, and exposure of microbiota-associated metabolites; host transformation and circulating exposure are not obligatory steps for all effects in distant organs. Circulating metabolite concentrations alone do not establish microbial production flux, target-tissue exposure, or target engagement. SCFAs, short-chain fatty acids; BBB, blood–brain barrier; TMAO, trimethylamine N-oxide; AD, Alzheimer's disease; LCA, lithocholic acid; DCA, deoxycholic acid; IPA, indole-3-propionic acid; mTOR, mechanistic target of rapamycin; CKD, chronic kidney disease; SAMP8, senescence-accelerated mouse prone 8; SAMR1, senescence-accelerated mouse resistant 1; APP/PS1, amyloid precursor protein/presenilin 1 transgenic mice; C2C12, mouse myoblast cell line.

## 6 Strategies for modulating microbial metabolic functions

### 6.1 Personalized nutrition based on microbial metabolic functions

Function-oriented personalized nutrition combines food structure with baseline gut microbiota and metabolic features to explore intervention selection. In a randomized crossover controlled-feeding study of healthy young adults, a diet rich in fiber and resistant starch, with larger food particles and less processing, increased surrogate measures of fecal bacterial biomass, fecal and serum short-chain fatty acid concentrations, and fecal energy excretion compared with a Western diet, while reducing the proportion of energy metabolizable by the host (89). This standardized dietary intervention demonstrated effects of food structure on microbial metabolic function and energy partitioning. Studies of intervention responses in older adults should also consider nutritional status, gastrointestinal transit, medication use, and organ function.

Substrate allocation and microbial interactions provide mechanistic clues. In a three-species system comprising *Bacteroides thetaiotaomicron, Escherichia coli*, and *Clostridium sporogenes, B. thetaiotaomicron* degraded pectin and supplied monosaccharides to *E. coli*, accompanied by reduced expression of the tryptophanase gene *tnaA* and lower indole production by *E. coli*. More tryptophan became available to *C. sporogenes*, increasing indolelactate, while indole-3-propionic acid showed an increasing trend. Fiber-associated reductions in indole were also observed in human fecal cultures and in mice colonized with fecal microbiota (90). These experiments show that substrate availability can alter metabolic output through microbial interactions and provide testable mechanistic leads for investigating differences in nutritional intervention responses.

Human trials and computational models have further explored response stratification. In a 6-month randomized trial involving 802 participants with prediabetes, the primary outcome—the percentage change in whole-blood HbA1c—did not differ significantly between the fiber-supplementation and usual-care groups. In a *post-hoc* analysis of four phenotypic subgroups, fasting glucose was lower in the fiber group than in its corresponding control group for phenotype 3, whereas glycated hemoglobin was lower in the fiber group than in its corresponding control group for phenotype 4. A response score based on baseline microbiota was subsequently evaluated for predictive performance in two previously conducted fiber-intervention cohorts (91). Another community-scale metabolic model predicted individual short-chain fatty acid production fluxes and compared predictions with measured output in *in vitro* and *ex vivo* culture systems; existing human data were used to examine associations between predicted values and blood measures (92). Prospective studies should compare interventions selected according to baseline features with non-stratified approaches to determine whether they provide additional functional or clinical benefits for older adults.

### 6.2 Metabolite supplementation and selective targeting of microbial enzymes

Metabolite supplementation can be used experimentally to test the functional effects of increased tissue exposure. After 1 month of receiving cyclodextrin-complexed lithocholic acid (LCA) in drinking water, 1.5-year-old mice had higher serum and muscle LCA concentrations and improved muscle mitochondrial respiration, grip strength, and exercise endurance. Muscle-specific AMPKα1/α2 deficiency significantly attenuated the improvements in grip strength and endurance (93). Another study used binding assays, genetic manipulation, and functional experiments to identify TULP3 as a mediator of LCA action and to characterize a mechanism involving sirtuin-related V1E1 deacetylation and AMPK activation (53). These studies respectively provide evidence on exposure and functional outcomes in aged mice and on target mechanisms in the corresponding experimental systems.

Selective inhibition of microbial enzymes alters metabolite exposure at the substrate-conversion step. In experiments, iodomethylcholine and fluoromethylcholine inhibited choline trimethylamine lyase CutC irreversibly and in a time-dependent manner and accumulated within gut microbes. Under the tested culture conditions for TMA-producing strains, both compounds inhibited TMA production without substantially affecting bacterial growth. Oral administration in mice lowered circulating TMAO, reduced choline-diet-induced platelet hyperreactivity and experimental thrombosis, and did not prolong tail bleeding time (94). This study supports an effect of inhibiting microbial precursor production on the corresponding thrombotic phenotype in mice.

Enzyme origin and substrate range affect inhibitor assessment. In mice with increased intestinal barrier permeability, microbial dipeptidyl peptidase 4 (DPP4) reduced active glucagon-like peptide-1 levels. Sitagliptin had limited activity against this microbial enzyme, whereas daurisoline-d4 inhibited its activity and improved glucose tolerance in diabetic mice (95). In another mouse experiment, a single dose of a covalent bile salt hydrolase (BSH) inhibitor reduced fecal BSH activity and deconjugated bile acid levels (96). An enzymatic study further found that some BSHs have acyltransferase activity that generates microbially conjugated bile acids, suggesting that assessments of inhibition should consider multiple substrates and products (97). In the broader context of aging interventions, adaptive-stress research indicates that effects depend on stimulus intensity, duration, and host status (98). These strategies are currently supported mainly by experimental studies. Their evaluation in older adults should consider dose and duration, target engagement, and metabolic exposure, as well as functional outcomes and safety.

### 6.3 Multi-omics stratification and prediction of intervention responses

Multi-omics stratification can be used to characterize metabolic states and their associations with risk. Wang et al. (99) classified 10,207 adults aged 40–93 years into five metabolic subtypes based on 21 metabolic measures and constructed a microbiome age index using 55 age-associated taxa identified in fecal metagenomes. Among participants aged 60 years or older, associations between metabolic subtypes and cardiovascular risk varied across strata of this index. A follow-up of a lifestyle intervention found that the metabolomics-estimated decrease in body mass index (BMI) was greater than the measured decrease, whereas the proteomics-estimated change was smaller (100). These studies describe risk associations and differences in changes across omics measures, respectively; the microbiome age index is not a direct measure of an individual's biological aging.

Baseline microbiota and metabolic features also provide leads for investigating nutritional intervention responses. In a single-arm, uncontrolled study, 27 participants with newly diagnosed diabetes or prediabetes received 10 g of inulin daily for 3 months. Changes in glycemic metabolism after the intervention were associated with baseline glycemic status, serum branched-chain amino acid derivatives and asparagine, and fecal indoles (101). These findings provide candidate response features in a disease context for evaluation in future stratification studies.

MICOM used metagenomic data from 186 Danish and Swedish individuals, including healthy controls and participants with type 1 or type 2 diabetes, to simulate short-chain fatty acid exchange fluxes under specified dietary constraints. Predicted bacterial growth rates were evaluated against replication rates inferred from sequencing data (102). McMLP integrated baseline microbiota, metabolomics, and dietary regimens to predict endpoint metabolite concentrations across data from six existing dietary-intervention studies and outperformed the algorithms against which it was compared (103). Prospective studies in older adults should test whether selecting interventions based on stratification or model outputs provides additional functional or clinical benefits over usual approaches. Related research tools and translational considerations are summarized in Table 2.

**Table 2:** Representative evidence and translational stages for modulating microbial metabolic functions and stratification.

Strategy or research tool	Target or prediction task	Representative study and population/model	Measured outcomes and evidence type	Translational stage and key question
Nutritional intervention and response stratification (exploratory)	Alters dietary substrate supply; explores baseline features associated with response.	Randomized crossover controlled-feeding study in healthy young adults (89); 6-month randomized fiber trial in 802 participants with prediabetes (91).	In the controlled-feeding study (89), a surrogate measure of fecal bacterial biomass, fecal and serum SCFA concentrations, and fecal energy excretion increased, while the proportion of host-metabolizable energy decreased. In the fiber trial (91), the primary outcome—the percentage change in whole-blood hemoglobin A1c (HbA1c)—did not differ significantly between groups; *post hoc* analyses found lower fasting glucose in subgroup 3 and lower HbA1c in subgroup 4 than in their respective control groups.	Human dietary intervention evidence; response stratification remains exploratory. Prospective studies should test whether selecting interventions using baseline features provides additional benefit.
Metabolite supplementation	Increases exposure to a specific metabolite to test its functional effects.	One-month lithocholic acid (LCA) supplementation in 1.5-year-old mice, with muscle-specific AMP-activated protein kinase (AMPK) experiments (93); separate LCA–Tubby-like protein 3 (TULP3) binding and function studies (53).	In (93), serum and muscle LCA concentrations, muscle mitochondrial respiration, grip strength, and endurance were measured; AMPKα1/α2 deficiency substantially attenuated improvements in grip strength and endurance. In (53), binding and functional experiments supported LCA–TULP3 involvement and sirtuin-related vacuolar ATPase subunit E1 (V1E1) deacetylation and AMPK activation.	Aged-animal and mechanistic evidence. Exposure, functional outcomes, and safety in older adults require evaluation.
Selective microbial enzyme inhibition: CutC	Inhibits microbial conversion of choline to TMA.	CutC inhibitor studies involving enzyme assays, TMA-producing strains, and mice (94).	TMA production was inhibited without an apparent reduction in bacterial growth under the tested strain culture conditions. In mice, circulating TMAO, platelet hyperreactivity, and experimental thrombosis decreased; tail bleeding time was not prolonged (94).	Preclinical evidence; human target engagement and durability remain to be tested.
Selective microbial enzyme inhibition: microbial DPP4	Selectively targets microbial dipeptidyl peptidase 4 (DPP4), distinguished from host DPP4.	Screening and mouse experiments, including models with increased gut barrier permeability and diabetic mice (95).	Daurisoline-d4 inhibited microbial DPP4 and improved glucose tolerance in diabetic mice; sitagliptin had limited activity against this microbial enzyme (95).	Preclinical evidence; microbial-target selectivity and functional outcomes in humans remain to be tested.
Selective bile salt hydrolase (BSH) inhibition	Inhibits bile acid deconjugation.	Covalent BSH inhibitor study in mice (96) and a separate BSH enzyme study (97).	A single dose lowered fecal BSH activity and deconjugated bile acid levels (96). Some BSH enzymes also showed acyltransferase activity that generates microbially conjugated bile acids (97).	Preclinical tool studies; effects on multiple substrates and products require evaluation.
Multi-omics stratification and candidate response features	Characterizes metabolic status and risk associations; explores baseline features associated with response.	Metabolic-subtype and microbiome-age study in 10,207 adults (99); single-arm inulin trial in 27 participants with newly diagnosed diabetes or prediabetes (101).	In (99), 21 metabolic measures were used to define five subtypes; among participants aged ≥60 years, associations between metabolic subtype and cardiovascular risk differed across microbiome-age strata. In (101), glycemic changes after 10 g/day inulin for 3 months were associated with baseline glucose and serum and fecal metabolites.	Population risk stratification and exploratory response analysis; prospective studies should test whether these tools can guide interventions.
Computational models	Simulates metabolic exchange fluxes; predicts endpoint metabolite levels after dietary interventions.	MICOM analysis using metagenomic data from 186 Danish and Swedish individuals, including healthy controls and participants with type 1 or type 2 diabetes (102); McMLP developed using data from six existing dietary intervention studies (103).	MICOM simulated SCFA exchange fluxes under specified dietary constraints and evaluated predicted bacterial growth rates against sequencing-inferred replication rates (102). McMLP predicted endpoint metabolite concentrations and outperformed the comparison algorithms (103).	Computational tools; independent validation and prospective testing of model-guided interventions for functional or clinical benefit are needed.

This table descriptively summarizes representative studies and translational stages; no formal evidence-quality grading was performed. Animal, cellular, and enzyme-study findings are limited to their experimental conditions. Model outputs are not equivalent to measured human metabolic activity or clinical benefit. Functional outcomes and safety in older adults require prospective evaluation.

## 7 Conclusion

Gut microbial metabolic remodeling provides a functional perspective for understanding age-related immunometabolic changes and organ phenotypes. Human studies have primarily identified observational associations between microbiota-associated metabolic features and barrier function, inflammation, and brain, liver, cardiovascular, and skeletal muscle phenotypes. Animal and cell studies support specific effects under defined experimental conditions. These findings should be interpreted in the context of relatively healthy older adults, people with frailty or multimorbidity, and patients with age-related diseases; findings from disease settings cannot be attributed directly to physiological aging. Microbial production, host biotransformation, circulating concentrations, tissue exposure, and target engagement also represent distinct levels of measurement.

Few human studies have assessed these processes together within the same study. Personalized nutrition, metabolite supplementation, selective inhibition of microbial enzymes, and multi-omics-based responder stratification remain exploratory research directions, with limited prospective evidence that they improve functional outcomes in older adults. Longitudinal studies that characterize health status and combine measurements of metabolite production and exposure with assessment of key confounders could clarify the temporal relationship between metabolic changes and functional outcomes. Prospective intervention studies incorporating functional or clinical endpoints could then test whether candidate strategies provide additional benefit and assess their safety.
